# Interfacial Engineering of V_2_O_5_ via Conductive Polyaniline for Accelerated Hydrogen Evolution Reaction

**DOI:** 10.3390/polym18111408

**Published:** 2026-06-05

**Authors:** Chaitany Jayprakash Raorane, Seong-Cheol Kim

**Affiliations:** School of Chemical Engineering, Yeungnam University, Gyeongsan 38541, Gyeongsanbuk-Do, Republic of Korea

**Keywords:** hydrogen evolution reaction, electropolymerization, polyaniline, charge transfer resistance

## Abstract

The hydrogen evolution reaction (HER) plays a pivotal role in electrochemical water splitting for sustainable hydrogen production. However, its practical implementation is hindered by sluggish kinetics and the reliance on costly noble-metal catalysts. In this work, a conductive polymer-inorganic hybrid electrode based on vanadium pentoxide (V_2_O_5_) and polyaniline (PANI) is rationally designed and fabricated on carbon cloth via a combined hydrothermal synthesis and electropolymerization strategy. Initially, hierarchical V_2_O_5_ nanoflowers were synthesized, followed by controlled PANI deposition through cyclic voltammetry at varying cycle numbers to tailor the interfacial architecture and electronic properties. Morphological and structural analyses reveal the formation of well-defined V_2_O_5_ nanoflowers uniformly decorated with PANI nanorods, establishing an interconnected conductive network. Among the prepared samples, the optimized V_2_O_5_-PANI-2 electrode exhibits superior interfacial integration and structural homogeneity. Electrochemical evaluation in 1.0 M KOH demonstrates that V_2_O_5_-PANI-2 achieves a low overpotential of 79.9 mV at −10 mA cm^−2^, accompanied by a small Tafel slope of 46.6 mV dec^−1^, indicating accelerated HER kinetics. Furthermore, the electrode shows reduced charge-transfer resistance and an enhanced electrochemically active surface area (ECSA), facilitating efficient charge transport and abundant active site exposure. The catalyst also delivers excellent durability, maintaining stable performance over 5000 CV cycles and prolonged 24 h operation. The enhanced HER performance is attributed to the synergistic interaction between V_2_O_5_ and the conductive PANI matrix, which promotes charge redistribution, improves electrical conductivity, and optimizes the adsorption/desorption energetics of hydrogen intermediates.

## 1. Introduction

The progress of clean and renewable energy resources is increasingly vital because of the rising global energy demands and the urgent requirement to reduce greenhouse gas emissions [1,2]. Hydrogen’s high energy density and the fact that its combustion generates only water as a by-product render it an effective energy carrier [3]. Electrocatalytic water splitting is recognized for its effectiveness and eco-friendliness among the various methods of hydrogen production. Nonetheless, the inadequate kinetics of the oxygen evolution reaction (OER) and the hydrogen evolution reaction (HER) present significant challenges to the widespread application of this method [4]. High-efficiency electrocatalysts can decrease energy usage in water electrolysis by enhancing reaction kinetics and minimizing the energy barrier [5]. At present, catalysts from the platinum (Pt) group, especially Pt/C and IrO_2_/RuO_2_, are mainly utilized in the production of hydrogen on an industrial scale. Nonetheless, the significant expense and restricted accessibility present considerable obstacles to expanding these technologies [6,7]. Consequently, the advancement of electrocatalysts based on transition metals is essential, since these substances not only exhibit potential performance but are also more cost-effective and plentiful for practical applications [8]. Thus, numerous research efforts have concentrated on the strategic design of various metal oxides to discover ideal candidates for enhancing HER performance.

Vanadium pentoxide (V_2_O_5_) has become a potential electrode material for various electrochemical applications due to its high activity and cost-effectiveness [9]. Significantly, V_2_O_5_ provides substantial benefits in electrochemical water splitting because of its natural structural stability and capacity to display various oxidation states, mainly V^5+^ and V^4+^, promoting effective redox reactions [10,11]. Additionally, V_2_O_5_ can facilitate HER kinetics by promoting the adsorption and desorption of hydrogen intermediates through its multivalent redox behavior and abundant surface V=O active sites [12,13]. Multiple synthesis approaches have been created to produce high-quality V_2_O_5_ nanostructures, such as hydrothermal routes [14], chemical vapor deposition techniques [15], spray pyrolysis methods [16], electrospinning techniques [17], and sol–gel route techniques [18]. Among these, hydrothermal synthesis is often favored because of it is easy to perform, affordable, and allows for superior control over morphology and crystallinity. In spite of these benefits, the direct application of bare V_2_O_5_ as an HER electrocatalyst typically shows restricted performance due to its low inherent electrical conductivity, inadequate electrochemically active sites, slow reaction kinetics, and quick charge recombination [19,20,21]. These constraints considerably impede effective electron flow and catalytic performance during HER. To address these limitations and improve the electrocatalytic efficiency of V_2_O_5_, different modification approaches have been investigated, including pairing with transition metal oxides/chalcogenides [22], combining with carbon-based materials [23], and conductive polymers [24]. For instance, Zn-doped vanadium oxide (Zn_0.4_V_1.6_O_5_) was developed to investigate the synergistic effect of transition-metal doping and oxygen vacancies, exhibiting an overpotential of 194 mV at a current density of 10 mA cm^−2^ in alkaline media [25]. In another study, a NiS_2_@V_2_O_5_/VS_2_ ternary heterojunction electrocatalyst was reported, delivering an overpotential of 216 mV at 10 mA cm^−2^, where the heterointerface played a crucial role in facilitating electron transport and catalytic activity [26]. Furthermore, a polypyrrole/V_2_O_5_/MnO_2_ hybrid electrocatalyst exhibited an HER overpotential of 192 mV at 10 mA cm^−2^, demonstrating the effectiveness of hybrid structure engineering for enhancing catalytic performance [24]. A carbon-dot-decorated silver-doped vanadium oxide (Ag/V_2_O_5_@C) demonstrated enhanced HER performance, requiring a low overpotential of 126 mV to achieve the same current density, owing to improved conductivity and charge-transfer characteristics [27].

Conductive polymers (CPs) have become highly effective supportive materials for a range of electrochemical energy conversion and storage applications [28,29,30]. Among the various conductive polymers, polyaniline (PANI) has garnered substantial interest because of its remarkable physicochemical characteristics, including straightforward synthesis, exceptional stability against environmental and chemical factors, high electrical conductivity, extensive surface area, minimal pore volume, and the utilization of inexpensive precursors. These benefits position PANI as one of the most promising materials for cutting-edge electrochemical systems [31,32]. Owing to these distinct features, PANI has been extensively applied in numerous technological fields, such as organic corrosion-resistant coatings [33], fuel cells [34], membranes [35], water splitting [36], and supercapacitors [32]. PANI is commonly used as a support material for electrocatalysts in devices related to electrochemical energy storage and conversion, including batteries, fuel cells, water splitting equipment and supercapacitors. Its simple preparation, high conductivity, affordable monomer source, and exceptional electrochemical stability greatly improve charge transfer, approachability of active sites, and overall performance of the device [31]. Recent studies have demonstrated that conductive PANI-based composite electrocatalysts exhibit promising HER activity in alkaline media. For instance, to improve the efficiency of electrochemical water splitting, a NiMnO_3_/PANI composite synthesized through a hydrothermal method exhibited an overpotential of 188 mV at a current density of −10 mA^−2^ [37]. Similarly, a NiFe_2_O_4_/PANI nanocomposite synthesized through a hydrothermal method achieved a low overpotential of 161 mV at a current density of −10 mA cm^−2^ [38]. In another study, PANI-coated nickel-cobalt phosphide nanowire arrays grown on nickel foam (NiCoP@PANI) exhibited excellent HER performance, requiring an overpotential of only 80.6 mV to deliver 10 mA cm^−2^ in 1.0 M KOH electrolyte [39]. Furthermore, a cobalt manganese oxide/polyaniline (CoMnO_3_/PANI) composite prepared via a hydrothermal route demonstrated an overpotential of 185 mV at 10 mA^−2^ [40].

In this work, a V_2_O_5_-PANI composite electrode was successfully developed on carbon cloth for enhanced HER in alkaline medium. Pristine V_2_O_5_ nanoflower structures were first synthesized through a hydrothermal method, followed by the electropolymerization of PANI with controlled deposition cycles to optimize the interfacial structure and catalytic performance. The conductive PANI layer was introduced to improve electrical conductivity, increase active site accessibility, and accelerate charge transfer kinetics. Among the prepared samples, the optimized V_2_O_5_-PANI-2 electrode exhibited superior HER activity with low overpotential, a small Tafel slope, reduced charge transfer resistance, and outstanding long-term stability.

## 2. Experimental Section

### 2.1. Chemicals

Ammonium metavanadate (NH_4_VO_3_ ≥ 99.0%) was acquired from Sigma-Aldrich, St. Louis, MO, USA. Anhydrous oxalic acid (C_2_H_2_O_4_, 98%) was obtained from Alfa Aesar, Gangnam-gu, Seoul, Republic of Korea. Aniline (C_6_H_5_NH_2_, 99.0%) and potassium hydroxide (KOH ≥ 85.0%) were provided by DaeJung Chemicals & Metals Co., Siheung-si, Republic of Korea. Sulfuric acid (H_2_SO_4_, ~95%) and hydrochloric acid (HCl, extra pure) was purchased from Duksan Reagents, Ansan-si, Republic of Korea. High-purity carbon cloth (CC), employed as the conductive substrate and current collector for electrode fabrication, was procured from NARA Cell-Tech Corporation, Seoul, Republic of Korea. All chemicals were of analytical grade and were used directly without any supplementary purification. Deionized (DI) water was used throughout all synthesis procedures and electrochemical experiments to ensure the consistency and purity of the reaction environment.

### 2.2. Synthesis of V_2_O_5_ and V_2_O_5_-PANI

Pristine V_2_O_5_ was directly synthesized on carbon cloth through a hydrothermal approach followed by thermal annealing. Initially, 10 mM of NH_4_VO_3_ was dissolved in 30 mL of ethanol under ultrasonication for 10 min to attain a homogeneous precursor solution. Subsequently, 3 mM of C_2_H_2_O_4_ was introduced into the solution to adjust the pH toward a mildly acidic condition and to facilitate precursor complexation. The resulting combination was again ultrasonicated for 10 min to ensure complete dissolution and uniform dispersion, followed by magnetic stirring for 30 min to achieve a stable reaction medium. The prepared precursor solution was then moved into a 50 mL Teflon-lined stainless-steel autoclave. Pre-cleaned carbon cloth, serving as the conductive substrate and current collector, was vertically immersed in the solution. The hydrothermal reaction was carried out at 180 °C for 24 h to promote the in situ growth of V_2_O_5_ nanostructures on the carbon cloth surface. After naturally cooling to room temperature, the obtained electrode was carefully washed with DI water to remove loosely attached particles and residual precursors, followed by drying at 70 °C for 8 h. The as-prepared sample was further annealed in air at 300 °C for 2 h.

**Figure 1:**
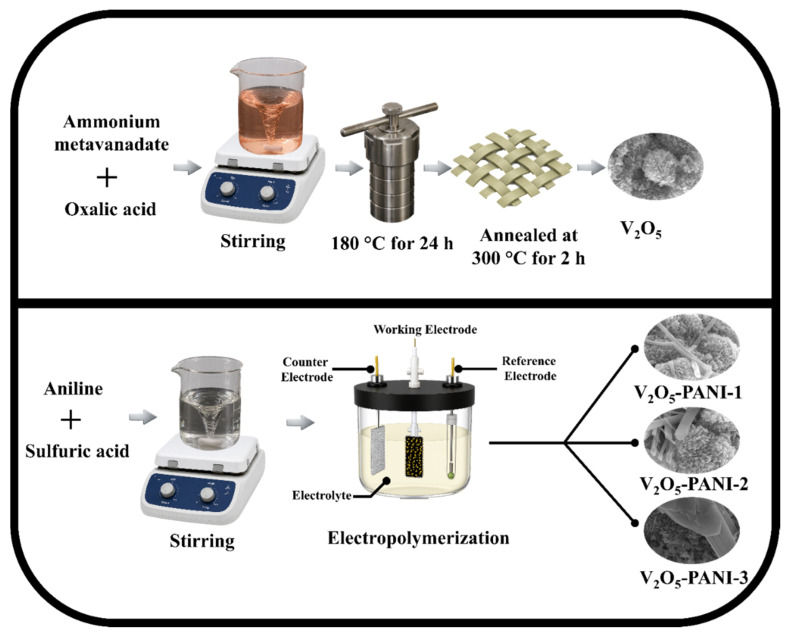
Schematic illustration of synthesis of V_2_O_5_ and V_2_O_5_-PANI electrocatalysts.

The synthesized V_2_O_5_ electrode was subsequently employed as the working electrode for the direct electropolymerization of polyaniline (PANI). The electrolyte for electropolymerization was prepared by adding 0.93 mL of aniline monomer into an aqueous acidic medium containing the required amount of H_2_SO_4_. The solution was continuously stirred until complete dispersion of the monomer was achieved, ensuring a uniform polymerization environment. Electrodeposition of PANI was performed using cyclic voltammetry (CV) in the potential window of 0 to 0.95 V at a scan rate of 50 mV s^−1^. Different PANI loadings were controlled by varying the number of CV deposition cycles, specifically 5, 10, and 15 cycles, to ensure uniform film growth and optimized surface coverage. The resulting electrodes were designated as V_2_O_5_-PANI-1, V_2_O_5_-PANI-2, and V_2_O_5_-PANI-3, respectively, which correspond to increasing PANI deposition levels. After electropolymerization, the deposited electrodes were thoroughly rinsed with DI water. The electrodes were then dried at 70 °C for 10 h. A schematic illustration of the overall hydrothermal synthesis and subsequent PANI electrodeposition process is presented in Figure 1.

### 2.3. Material Characterization

The crystallographic structure and phase conformation of the prepared materials were examined using powder X-ray diffraction (XRD) with an X’Pert Pro diffractometer (PANalytical, Almelo, The Netherlands) employing Cu Kα radiation (λ = 1.5418 Å). Surface chemical composition, elemental valence states, and interfacial bonding characteristics were investigated by X-ray photoelectron spectroscopy (XPS) using a Thermo Scientific K-Alpha system (Cheshire, UK analysis system). The surface architecture and morphological characteristics of the electrodes were examined using field-emission scanning electron microscopy (FESEM) on a HITACHI S-4800 instrument (FESEM, HITACHI S-4800, Tokyo, Japan). Furthermore, the field-emission scanning electron microscopy (FESEM, HITACHI S-4800, Tokyo, Japan) system coupled with energy-dispersive X-ray spectroscopy (EDX) was used to regulate the elemental composition and to obtain elemental mapping images.

### 2.4. Electrochemical Analysis

All electrochemical measurements were performed at room temperature using a BioLogic VSP-300 electrochemical workstation, Gières, France, in a standard three-electrode system with 1.0 M KOH solution as the electrolyte. The active materials were directly grown on pre-cleaned carbon cloth, which functioned as both the working electrode substrate and current collector. Before synthesis, the carbon cloth was ultrasonically cleaned in 1 M HCl, deionized water, and ethanol for 20 min each, followed by overnight drying at 60 °C. An Ag/AgCl electrode and a Pt plate were used as the reference and counter electrodes, respectively. Linear sweep voltammetry was recorded at 5 mV s^−1^ to evaluate the HER activity of the prepared catalysts, and all potentials were converted to the reversible hydrogen electrode scale for accurate comparison. The electrochemical double-layer capacitance was obtained from cyclic voltammetry measurements conducted in the non-Faradaic region of 0.1–0.2 V vs. Ag/AgCl at scan rates of 5–25 mV s^−1^. The electrochemically active surface area was then estimated from the C_dl_ values using a specific capacitance, C_s_, of 0.040 mF cm^−2^ [41,42]:

ECSA = C_dl_/C_s_ (1)

Electrochemical impedance spectroscopy (EIS) was performed from 100 kHz to 0.1 Hz with a 10 mV AC amplitude to examine charge-transfer behavior at the electrode/electrolyte interface. The durability of the optimized electrocatalyst was evaluated using 5000 continuous CV cycles at 50 mV s^−1^, followed by comparison of LSV curves before and after cycling. Long-term stability was further assessed by chronopotentiometry at a constant current density under continuous HER operation.

## 3. Results and Discussion

**Figure 2:**
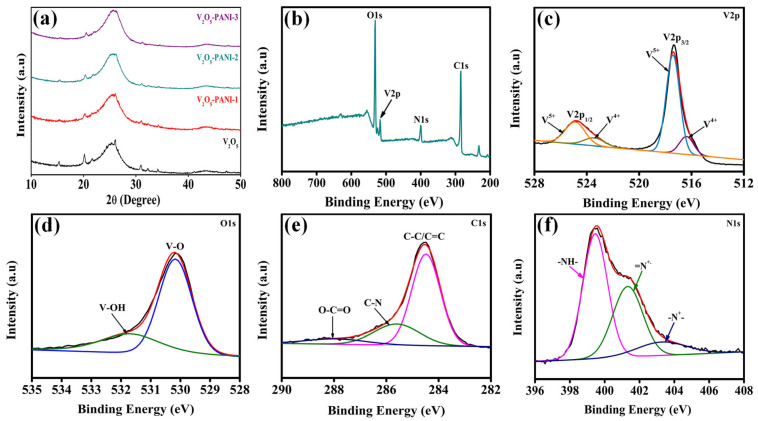
(**a**) XRD spectra of all the electrocatalysts, (**b**) XPS survey spectra, and high-resolution XPS spectra of (**c**) V2p, (**d**) O 1s, (**e**) C1s, and (**f**) N1s of V_2_O_5_-PANI-2 composite.

The X-ray diffraction (XRD) patterns presented in Figure 2a for the hydrothermally synthesized V_2_O_5_ and electrodeposited V_2_O_5_-PANI composite exhibit distinct diffraction peaks within the 2θ range of 10–50°. The prominent diffraction peaks located at 2θ values of 15.3°, 20.1°, 21.6°, 26.0°, 31.1°, 32.2°, 33.2°, 34.1°, and 41.1° were indexed to the (200), (001), (101), (110), (400), (011), (111), (310), and (002) crystal planes, respectively, confirming the successful formation of the V_2_O_5_ phase. These peaks are characteristic of the orthorhombic crystal phase of V_2_O_5_ and are in good agreement with the standard JCPDS card No. 00-009-0387 [43,44]. The presence of sharp and well-resolved diffraction peaks indicates the high crystallinity and well-developed crystal structure of the synthesized V_2_O_5_. In addition, two broad diffraction peaks observed at 2θ values of 25.2° and 43.3° were assigned to the (002) and (100) planes of carbon cloth, respectively, which are consistent with JCPDS card No. 75-1621 [45]. For the V_2_O_5_-PANI composite electrodes, no distinct crystalline peaks corresponding to PANI were observed in the XRD patterns. This behavior suggests that the deposited PANI mainly exists in an amorphous form [45,46]. Interestingly, with increasing PANI deposition cycles, the intensity of the broad diffraction peak at 25.2° gradually increased, further supporting the successful formation of amorphous PANI on the electrode surface. Simultaneously, the characteristic diffraction peak intensities of V_2_O_5_ showed a gradual decrease with increasing PANI loading. This reduction in peak intensity indicates that the PANI layer progressively covered the crystalline V_2_O_5_ surface, resulting in partial attenuation of the V_2_O_5_ diffraction signals. These observations collectively confirm the successful deposition of PANI onto the V_2_O_5_ framework and the effective formation of the V_2_O_5_-PANI composite structure.

The surface chemical composition and oxidation states of the V_2_O_5_-PANI-2 composite were analyzed using X-ray photoelectron spectroscopy (XPS). The full XPS survey spectrum (Figure 2b) confirms the presence of vanadium (V), oxygen (O), carbon (C), and nitrogen (N) elements within the composite. To further elucidate the chemical states of these elements, the XPS spectra were deconvoluted; the high-resolution spectra of V2p, O1s, C1s, and N1s are presented in Figure 2. The high-resolution V2p spectrum shown in Figure 2c confirms the coexistence of mixed vanadium oxidation states, namely V^4+^ and V^5+^. The deconvoluted peaks located at 516.3 eV and 523.4 eV are assigned to the V^4+^ species corresponding to the V2p_3/2_ and V2p_1/2_ spin–orbit components, respectively. In addition, the dominant peaks observed at 517.3 eV and 524.8 eV are attributed to the V^5+^ oxidation state in the V2p_3/2_ and V2p_1/2_ regions, respectively [43,47]. The O1s spectrum presented in Figure 2d consists of two major components. The dominant peak centered at 530.1 eV is assigned to lattice oxygen associated with the V-O bond in the V_2_O_5_ crystal framework, confirming the formation of metal-oxygen coordination. A second weaker peak located at 531.7 eV corresponds to surface hydroxyl species (V-OH) or adsorbed oxygen-containing groups, which may originate from surface adsorption during synthesis or exposure to ambient conditions [22,43,48]. The high-resolution C1s spectrum shown in Figure 2e confirms the successful incorporation of PANI on the V_2_O_5_ surface. The main peaks at 284.4, 285.5, and 288.1 eV are assigned to C-C/C=C, C-N, and O-C=O bonds, respectively [49,50]. The N1s spectrum displayed in Figure 2f provides direct evidence for the successful electropolymerization of PANI on the V_2_O_5_ surface. The broad peak around 399 eV was deconvoluted into three distinct nitrogen species located at 399.4, 401.3, and 403.3 eV. The peak located at 399.4 eV is attributed to benzenoid amine nitrogen (-NH-), which is present in the polyaniline backbone. The peak centered at 401.3 eV corresponds to nitrogen cationic radicals (=N^+**.**^), which are associated with the oxidized conductive state of PANI. In addition, the higher binding energy peak observed at 403.3 eV is assigned to protonated amine nitrogen (-N^+^-), indicating protonation of the polymer chain and enhanced electrical conductivity of the composite electrode. [51,52,53]. The presence of these nitrogen functionalities confirms the successful deposition of conductive PANI and suggests strong electronic interaction between PANI and V_2_O_5_, which contributes to improved HER performance.

**Figure 3:**
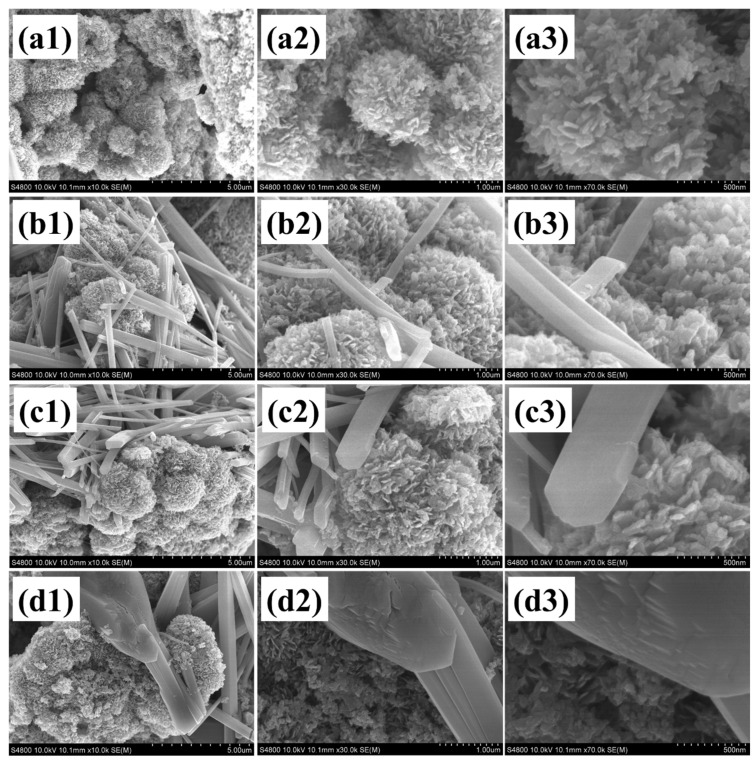
FESEM micrograph images of (**a1**–**a3**) V_2_O_5_, (**b1**–**b3**) V_2_O_5_-PANI-1, (**c1**–**c3**) V_2_O_5_-PANI-2, and (**d1**–**d3**) V_2_O_5_-PANI-3 electrocatalysts.

The surface morphology and microstructural evolution of pristine V_2_O_5_ and the V_2_O_5_-PANI composite electrodes were investigated using field-emission scanning electron microscopy (FESEM), and the corresponding images are presented in Figure 3. The pristine V_2_O_5_ sample, shown in Figure 3(a1–a3), exhibits a well-defined nanoflower-like architecture composed of densely packed and interconnected nanosheets. After electropolymerization of PANI, significant morphological changes were observed in the composite electrodes. For V_2_O_5_-PANI-1 (Figure 3(b1–b3)), corresponding to 5 CV deposition cycles, the nanoflower structure of V_2_O_5_ remains clearly visible, while the initial growth of PANI nanorods can be observed on the surface. These rod-like structures are sparsely distributed and partially cover the V_2_O_5_ nanoflowers, indicating the early stage of PANI deposition. Although the conductive polymer improves surface conductivity, the relatively low PANI loading may limit the number of electrochemically active sites available for the HER. In the case of V_2_O_5_-PANI-2, presented in Figure 3(c1–c3), which was prepared with 10 CV deposition cycles, a more uniform and optimized hybrid structure is formed. The PANI nanorods are homogeneously distributed over the V_2_O_5_ nanoflower framework without causing severe aggregation or blocking of the active surface. The intimate interfacial contact between the nanoflower-like V_2_O_5_ and the conductive PANI nanorods creates a highly interconnected network that significantly enhances electron transport, improves electrolyte accessibility, and increases the density of catalytically active sites. For V_2_O_5_-PANI-3 (Figure 3(d1–d3)), synthesized using 15 CV deposition cycles, excessive PANI deposition leads to the formation of thicker and more densely packed nanorod structures, resulting in partial agglomeration and over coverage of the V_2_O_5_ nanoflowers. This excessive polymer layer can hinder electrolyte diffusion and block the accessibility of the intrinsic active sites of V_2_O_5_, thereby reducing catalytic efficiency. Among all samples, V_2_O_5_-PANI-2 exhibits the most desirable microstructure with an optimal balance between active site exposure, conductivity enhancement, and interfacial charge transport, which directly supports its superior HER performance.

**Figure 4:**
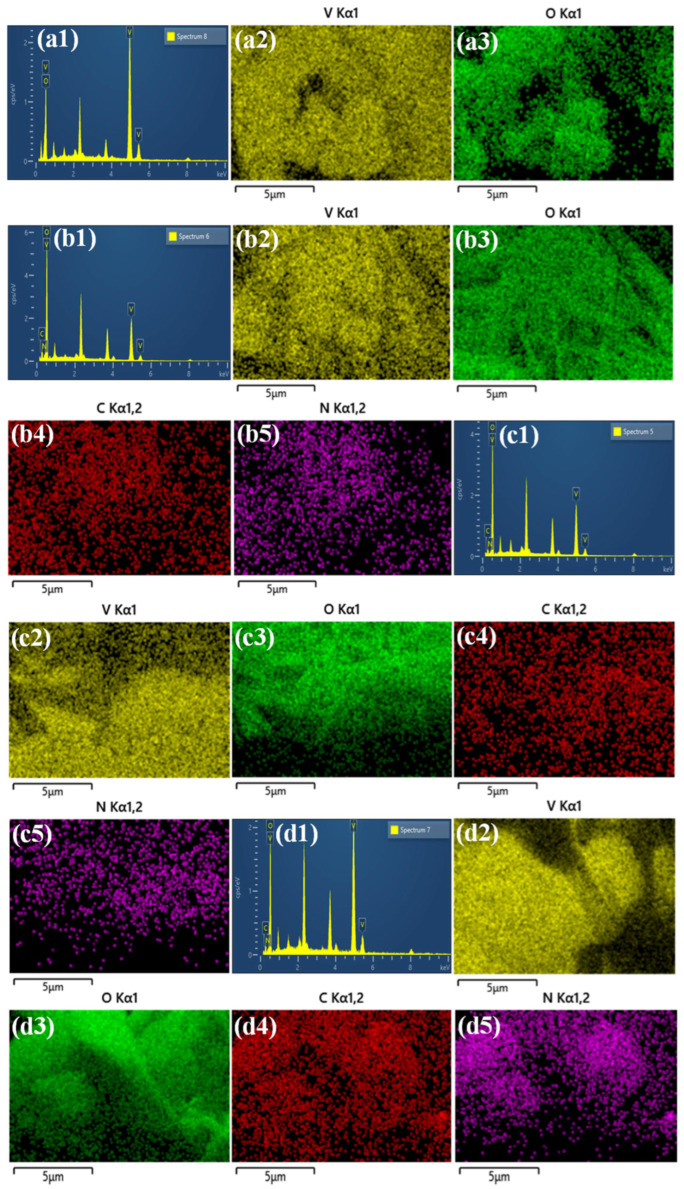
Energy-dispersive X-ray spectroscopy analysis and elemental mapping data of (**a1**–**a3**) V_2_O_5_, (**b1**–**b5**) V_2_O_5_-PANI-1, (**c1**–**c5**) V_2_O_5_-PANI-2, and (**d1**–**d5**) V_2_O_5_-PANI-3.

The elemental composition and surface distribution of pristine V_2_O_5_ and the V_2_O_5_-PANI composite were further investigated using energy-dispersive X-ray spectroscopy (EDX), and the corresponding spectra and elemental mapping images are presented in Figure 4. For pristine V_2_O_5_, the EDX spectrum shown in Figure 4(a1) confirms the presence of only V and O, with weight percentages of 62.22 wt% and 37.78 wt%, respectively, indicating the successful formation of pure vanadium oxide without detectable impurity phases. The corresponding elemental mapping images in Figure 4(a2,a3) demonstrate a uniform spatial distribution of V and O throughout the analyzed region, confirming the homogeneous growth of V_2_O_5_ over the carbon cloth substrate. After electropolymerization of PANI, additional signals corresponding to C and N become clearly visible in the EDX spectra, confirming the successful deposition of the conductive polymer layer. For V_2_O_5_-PANI-1 (Figure 4(b1)), the composition consists of 54.60 wt% V, 23.81 wt% O, 20.50 wt% C, and 1.09 wt% N. The elemental mapping images in Figure 4(b2–b5) show that V, O, C, and N are uniformly distributed, confirming good interfacial contact between the oxide and polymer phases. For the V_2_O_5_-PANI-2 sample (Figure 4(c1)), the EDX results reveal a balanced composition of 53.29 wt% V, 23.23 wt% O, 21.25 wt% C, and 2.23 wt% N. This optimized elemental ratio suggests an ideal PANI loading that provides sufficient conductive coverage without excessive blockage of the intrinsic V_2_O_5_ active sites. The elemental mapping images shown in Figure 4(c2–c5) demonstrate a highly uniform dispersion of all constituent elements, indicating strong interfacial integration and effective hybridization between V_2_O_5_ and PANI. In contrast, V_2_O_5_-PANI-3 (Figure 4(d1)) exhibits a further increase in carbon and nitrogen content, with a composition of 50.46 wt% V, 20.80 wt% O, 23.73 wt% C, and 5.01 wt% N. The higher nitrogen content indicates excessive PANI deposition, which is consistent with the FESEM observations of dense polymer overgrowth. Although the conductivity may improve, excessive PANI coverage can hinder electrolyte diffusion and reduce the exposure of catalytically active V_2_O_5_ sites. The mapping images in Figure 4(d2–d5) still show uniform elemental dispersion; however, the thicker polymer coverage may negatively influence catalytic efficiency by limiting direct access to the oxide surface.

**Figure 5:**
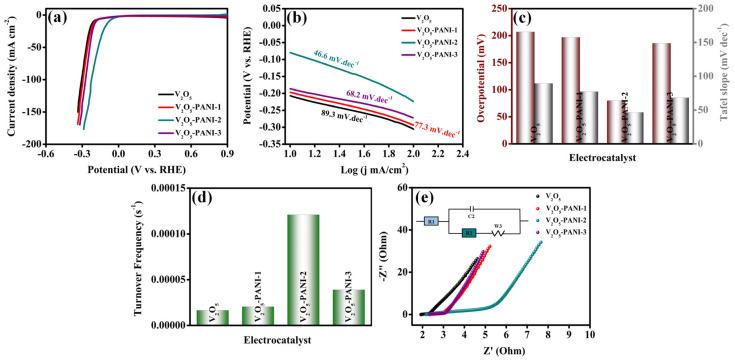
HER performances of all the electrocatalyst: (**a**) LSV curves at 5 mV/s scan rate, (**b**) equivalent Tafel slopes, (**c**) HER performance concerning overpotential at −10 mA cm^−2^ and Tafel slope, (**d**) TOF plot, and (**e**) EIS spectra (inset equivalent circuit) of all the electrocatalysts.

The electrocatalytic HER performance of the as-prepared catalysts was investigated in 1.0 M KOH electrolyte, and the corresponding electrochemical results are presented in Figure 5. All polarization curves were carefully calibrated to the RHE scale to ensure an accurate evaluation of the intrinsic catalytic activity of the electrodes. Figure 5a,c present the comparative HER polarization behavior of pristine V_2_O_5_ and the V_2_O_5_-PANI composite catalysts, namely V_2_O_5_-PANI-1, V_2_O_5_-PANI-2, and V_2_O_5_-PANI-3. Among all investigated samples, pristine V_2_O_5_ exhibited the highest overpotential of 207.0 mV at a current density of −10 mA cm^−2^, indicating relatively sluggish HER kinetics. Although V_2_O_5_ inherently provides redox-active sites that can participate in hydrogen evolution, its limited electrical conductivity and slower interfacial electron transfer significantly restrict its catalytic efficiency. Upon electro polymerization of PANI, a substantial enhancement in HER activity was observed for all composite catalysts. Specifically, V_2_O_5_-PANI-1, V_2_O_5_-PANI-2, and V_2_O_5_-PANI-3 exhibited significantly reduced overpotentials of 196.8, 79.9, and 186.0 mV, respectively, at the same current density of −10 mA cm^−2^. This remarkable improvement confirms the strong synergistic interaction between the V_2_O_5_ framework and the conductive PANI network, which effectively promotes charge transport and increases the accessibility of catalytically active sites. Among the series, V_2_O_5_-PANI-2 demonstrated the best HER performance with the lowest overpotential of 79.9 mV, indicating its optimized catalytic configuration. This superior activity can be attributed to the ideal PANI loading and its uniform distribution over the V_2_O_5_ surface, which collectively enhance electrical conductivity, facilitate rapid electron migration, and expose a larger number of active reaction centers. To further evaluate the HER kinetics, Tafel slope analysis was performed using the polarization data, as shown in Figure 5b,c. The V_2_O_5_-PANI-2 electrocatalyst exhibited the lowest Tafel slope of 46.6 mV dec^−1^, indicating the most favorable reaction kinetics and faster hydrogen adsorption–desorption processes. The Tafel slope values followed the order of V_2_O_5_-PANI-2 (46.6 mV dec^−1^) < V_2_O_5_-PANI-3 (68.2 mV dec^−1^) < V_2_O_5_-PANI-1 (77.3 mV dec^−1^) < V_2_O_5_ (89.3 mV dec^−1^). The significantly lower Tafel slope of V_2_O_5_-PANI-2 suggests that the HER proceeds more efficiently on this catalyst surface, with reduced kinetic barriers and accelerated charge-transfer processes. The Tafel slope is an important kinetic parameter for understanding the dominant reaction pathway of the HER. According to established electrochemical theory, the HER mechanism can be interpreted based on the obtained Tafel slope values. A Tafel slope in the range of 30–40 mV dec^−1^ generally indicates that the reaction proceeds through the Volmer-Tafel pathway, where the recombination of two adsorbed hydrogen intermediates (H*) is the rate-determining step [54,55].

The initial water dissociation and hydrogen adsorption process can be represented by the Volmer reaction:

H_2_O + e^−^ → H* + OH^−^ (Volmer reaction) (2)

followed by the Tafel recombination step:

H* + H* → H_2_ (Tafel reaction) (3)

On the other hand, Tafel slope values between 40 and 120 mV dec^−1^ suggest that the HER predominantly follows the Volmer-Heyrovsky mechanism, in which electrochemical desorption of adsorbed hydrogen acts as the rate-determining step [56]. This process is represented by the Heyrovsky reaction:

H* + H_2_O + e^−^ → H_2_ + OH^−^ (Heyrovsky reaction) (4)

For the V_2_O_5_-PANI-2 electrocatalyst, the experimentally obtained Tafel slope of 46.6 mV dec^−1^ indicates that the HER mainly proceeds through the Volmer-Heyrovsky mechanism. This result suggests that the electrochemical desorption step governs the overall reaction kinetics, while the optimized interfacial interaction between V_2_O_5_ and the conductive PANI network facilitates rapid charge transfer and efficient hydrogen intermediate conversion, thereby enhancing HER performance. The intrinsic catalytic activity of the prepared electrodes was further evaluated by calculating the turnover frequency (TOF), which represents the number of hydrogen molecules generated per active site per second during the HER. The TOF values were estimated under anodic peak conditions, assuming that each electrochemically accessible metal center participates as an active catalytic site. The calculated TOF values for V_2_O_5_, V_2_O_5_-PANI-1, V_2_O_5_-PANI-2, and V_2_O_5_-PANI-3 were 1.64 × 10^−5^, 2.04 × 10^−5^, 1.21 × 10^−4^, and 3.89 × 10^−5^ s^−1^, respectively, as presented in Figure 5d. Among all the investigated samples, V_2_O_5_-PANI-2 exhibited the highest TOF value, indicating its superior intrinsic catalytic activity for the HER. This optimized electronic environment promotes efficient hydrogen intermediate adsorption (H*) and conversion, thereby enhancing HER kinetics and overall electrocatalytic activity [57]. Electrochemical impedance spectroscopy (EIS) was further employed to investigate the interfacial charge transfer characteristics during HER, and the corresponding Nyquist plots are shown in Figure 5e. The EIS data were fitted using an equivalent circuit model shown in inset figure, to quantitatively analyze the electrochemical resistance components. In the Nyquist plots, the diameter of the semicircle in the high-frequency region corresponds to the charge transfer resistance (R_ct_), which directly reflects the efficiency of electron transfer at the electrode-electrolyte interface [58]. Notably, V_2_O_5_-PANI-2 exhibited the smallest semicircle and the lowest Rct value of 3.2 Ω, demonstrating the fastest interfacial electron transfer and the most efficient HER kinetics among all samples. In comparison, pristine V_2_O_5_ showed a significantly higher R_ct_ value of 11.7 Ω, while V_2_O_5_-PANI-1 and V_2_O_5_-PANI-3 displayed intermediate values of 9.6 and 6.3 Ω, respectively. The substantially reduced R_ct_ value of V_2_O_5_-PANI-2 clearly indicates that the optimized incorporation of PANI effectively improves the electrical conductivity and interfacial charge transport behavior of the composite, thereby facilitating rapid electron injection and enhanced catalytic activity for hydrogen evolution.

**Figure 6:**
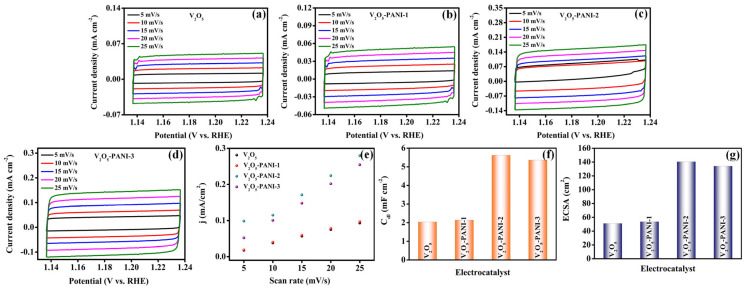
Cyclic voltammetry analysis at different scan rates: (**a**) V_2_O_5_, (**b**) V_2_O_5_-PANI-1, (**c**) V_2_O_5_-PANI-2, and (**d**) V_2_O_5_-PANI-3. (**e**) 2C_dl_ graph, (**f**) C_dl_ graph, and (**g**) ECSA graph of all the electrocatalysts.

The CV analysis of all the electrocatalysts were evaluated to investigate their surface electrochemical behavior and estimate the density of accessible active sites. Figure 6a–d presents the CV curves of pristine V_2_O_5_ and the V_2_O_5_-PANI composite catalysts recorded at various scan rates within the non-faradaic potential region, along with the corresponding current density versus potential profiles. A gradual increase in current response with increasing scan rate was clearly observed for all samples, which is characteristic of capacitive behavior and confirms the strong dependence of electrochemical activity on surface charge accumulation processes. To further understand the relationship between catalytic performance and the number of exposed active sites, the electrochemically active surface area (ECSA) was estimated from the double-layer capacitance (C_dl_). The 2C_dl_ values (Figure 6e) were obtained by plotting the difference in anodic and cathodic current densities against the scan rate, where the slope of the linear fitting corresponds to the double-layer capacitance. Since C_dl_ is directly proportional to the available electrochemically active surface, it serves as an important indicator of catalytic site density and surface accessibility. The calculated C_dl_ values, shown in Figure 6f, for pristine V_2_O_5_, V_2_O_5_-PANI-1, V_2_O_5_-PANI-2, and V_2_O_5_-PANI-3 were 2.04, 2.14, 5.62, and 5.36 mF cm^−2^, respectively. Based on these values, the corresponding ECSA values were determined to be 51.12, 53.62, 140.50, and 134.12 cm^2^, respectively, as summarized in Figure 6g. Among all the catalysts, V_2_O_5_-PANI-2 exhibited the highest C_dl_ and ECSA values, indicating the largest electrochemically accessible surface area and the highest density of exposed active sites. The significant enhancement in both C_dl_ and ECSA after the incorporation of PANI demonstrates that the conductive polymer effectively modifies the surface architecture of V_2_O_5_, leading to improved interfacial contact, enhanced electrolyte penetration, and greater exposure of catalytically active centers.

**Figure 7:**
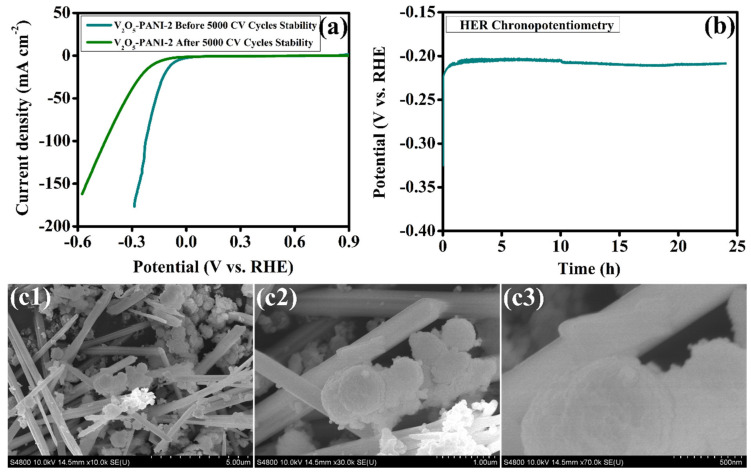
(**a**) LSV curves of V_2_O_5_-PANI-2 before and after 5000 CV cycles. (**b**) Chronopotentiometry analysis. (**c1**–**c3**) After stability FESEM images of V_2_O_5_-PANI-2 electrocatalyst.

A comparative assessment of the electrocatalytic performance of V_2_O_5_-PANI-2 with recently reported HER electrocatalysts is presented in Table 1. The summarized electrochemical parameters demonstrate the competitive HER activity of the developed catalyst, highlighting the effectiveness of the V_2_O_5_-PANI hybrid architecture for alkaline hydrogen evolution. The long-term electrochemical durability of the optimized V_2_O_5_-PANI-2 electrocatalyst was systematically evaluated under both dynamic and steady-state operating conditions to assess its structural and catalytic stability during the HER. To examine the durability under repeated redox cycling, accelerated stability testing was performed using continuous CV scanning for 5000 cycles. As presented in Figure 7a, the polarization curve of V_2_O_5_-PANI-2 after prolonged cycling shows an observable shift toward a higher overpotential region, indicating a certain degree of catalytic performance degradation. Specifically, the overpotential required to achieve a current density of −10 mA cm^−2^ increased to 180.7 mV compared to its initial value, suggesting partial deterioration of the catalytic interface during repeated electrochemical operation. This decline in HER activity can be attributed to repeated polarization, which may induce slight detachment of the active material from the substrate or changes in the conductive PANI network, which can negatively influence charge transfer efficiency. To further evaluate the operational stability under practical working conditions, chronopotentiometric analysis was carried out at a constant current density of −10 mA cm^−2^ for 24 h, as shown in Figure 7b. Unlike the CV cycling results, the potential-time profile during the long-term constant-current test exhibited a gradual decrease after several hours of operation, indicating an activation behavior rather than continuous degradation. These results demonstrate that although slight performance loss is observed under harsh accelerated CV cycling, the V_2_O_5_-PANI-2 electrocatalyst exhibits reasonable stability under practical continuous HER operation conditions. The post-stability FESEM images of V_2_O_5_-PANI-2 after 5000 continuous CV cycles (Figure 7(c1–c3)) reveal that the rod-like PANI structures remain largely preserved, indicating good structural stability of the conductive polymer framework under repeated electrochemical cycling. However, the V_2_O_5_ nanoflower-like structures show partial agglomeration and surface reconstruction, which may be attributed to repeated redox polarization and localized structural rearrangement during prolonged cycling. These morphological changes likely contribute to the increase in overpotential observed after the durability test.

## 4. Conclusions

In conclusion, a series of V_2_O_5_-PANI composite electrodes were successfully fabricated on carbon cloth through hydrothermal synthesis followed by controlled electropolymerization for efficient HER in alkaline medium. Pristine V_2_O_5_ exhibited a nanoflower-like morphology, while the electrodeposited PANI formed nanorod-like structures that effectively improved the surface conductivity and interfacial charge transfer. Among the prepared samples, V_2_O_5_-PANI-2 showed the most optimized architecture with uniform PANI distribution and strong interfacial interaction between V_2_O_5_ and the conductive polymer matrix. Electrochemical results demonstrated that V_2_O_5_-PANI-2 delivered the best HER performance with a low overpotential of 79.9 mV at −10 mA cm^−2^, a small Tafel slope of 46.6 mV dec^−1^, and the lowest charge transfer resistance of 3.2 Ω. In addition, the catalyst exhibited the highest double-layer capacitance of 5.62 mF cm^−2^ and an electrochemically active surface area of 140.50 cm^2^, confirming the presence of abundant accessible active sites. The electrode demonstrated reasonable electrochemical stability after 5000 CV cycles and sustained catalytic activity during 24 h chronopotentiometric testing. These findings confirm that the synergistic integration of V_2_O_5_ and PANI is an effective strategy to enhance HER activity and stability, offering a promising pathway for the development of cost-effective and high-performance electrocatalysts for sustainable hydrogen production.

## Figures and Tables

**Table 1:** Comparison of HER results of developed electrocatalyst and other reported electrocatalysts.

Electrocatalyst	Electrolyte	Overpotential (mV @ 10 mA cm^−2^)	Ref.
CdS-V_2_O_5_/g-C_3_N_4_	1 M KOH	202	[23]
PPy/V_2_O_5_/MnO_2_	1 M KOH	192	[24]
NiS_2_@V_2_O_5_/VS_2_	1 M KOH	216	[26]
NiMnO_3_/PANI	1 M KOH	188	[37]
NiFe_2_O_4_/PANI	1 M KOH	161	[38]
NiCoP@PANI	1 M KOH	80.6	[39]
CoMnO_3_/PANI	1 M KOH	185	[40]
V_2_O_5_	1 M KOH	177	[43]
VO/CoN	1 M KOH	80.5	[59]
NdCoO_3_/PANI	1 M KOH	113	[60]
V_2_O_5_-PANI-2	1 M KOH	79.9	Present work

## Data Availability

The original contributions presented in this study are included in the article. Further inquiries can be directed to the corresponding author.

## References

[1] Vinodh R., Kalanur S.S., Natarajan S.K., Pollet B.G. (2023). Recent advancements of polymeric membranes in anion exchange membrane water electrolyzer (AEMWE): A critical review. Polymers.

[2] Bhosale M., Baby N., Magdum S.S., Murugan N., Kim Y.A., Thangarasu S., Oh T.-H. (2024). Hierarchical nanoassembly of Ni_3_S_2_-MoS_2_ interconnected with CeO_2_ as a highly remarkable hybrid electrocatalyst for enhancing water oxidation and energy storage. J. Energy Storage.

[3] Bhuiyan M.M.H., Siddique Z. (2025). Hydrogen as an alternative fuel: A comprehensive review of challenges and opportunities in production, storage, and transportation. Int. J. Hydrogen Energy.

[4] Qian Q., Zhu Y., Ahmad N., Feng Y., Zhang H., Cheng M., Liu H., Xiao C., Zhang G., Xie Y. (2024). Recent advancements in electrochemical hydrogen production via hybrid water splitting. Adv. Mater..

[5] Wu Y., Chen P. (2026). Hybrid water electrolysis toward energy-efficient hydrogen production coupled with value-added chemical synthesis. Green Chem..

[6] Lin B.-L., Chen X., Niu B.-T., Lin Y.-T., Chen Y.-X., Lin X.-M. (2024). The research progress of ruthenium-based catalysts for the alkaline hydrogen evolution reaction in water electrolysis. Catalysts.

[7] Gao H., Ma Z., Wang C., Zhang L., Zhang Y. (2026). Modification strategies of metal organic framework-derived tungsten-based electrocatalysts for hydrogen evolution. J. Alloys Compd..

[8] Zhang X., Liu Y., Zeng Z., Zou Y., Wang W., Zhang J., Wang J., Kong X., Meng X. (2025). Core–Shell CoS_2_/FeS_2_ Heterojunction Encapsulated in N-Doped Carbon Nanocubes Derived from Coordination Polymers for Electrocatalytic Alkaline Water/Seawater Splitting. Polymers.

[9] Temam A.G., Alshoaibi A., Getaneh S.A., Awada C., Nwanya A.C., Ejikeme P.M., Ezema F.I. (2023). Recent progress on V_2_O_5_ based electroactive materials: Synthesis, properties, and supercapacitor application. Curr. Opin. Electrochem..

[10] Zhao X., Yan Y., Mao L., Fu M., Zhao H., Sun L., Xiao Y., Dong G. (2018). A relationship between the V^4+^/V^5+^ ratio and the surface dispersion, surface acidity, and redox performance of V_2_O_5_–WO_3_/TiO_2_ SCR catalysts. RSC Adv..

[11] Hu P., Hu P., Vu T.D., Li M., Wang S., Ke Y., Zeng X., Mai L., Long Y. (2023). Vanadium oxide: Phase diagrams, structures, synthesis, applications. Chem. Rev..

[12] Munawar T., Bashir A., Nadeem M.S., Mukhtar F., Manzoor S., Ashiq M.N., Khan S.A., Koc M., Iqbal F. (2024). Scalable synthesis of MOF-derived Nd_2_O_3_@ C and V_2_O_5_@ C nanohybrid: Efficient electrocatalyst for OER in alkaline medium. Fuel.

[13] Zhang J., Zhang H., Liu M., Xu Q., Jiang H., Li C. (2020). Cobalt-stabilized oxygen vacancy of V_2_O_5_ nanosheet arrays with delocalized valence electron for alkaline water splitting. Chem. Eng. Sci..

[14] Magdum S.S., Palanisamy G., Selvakumar K., Thangarasu S., Oh T.H. (2025). Multicomponent nanostructured catalyst of rGO-bi-V_2_O_5_ for photocatalytic degradation of methylene blue and supercapacitor applications. FlatChem.

[15] George A., Raj A.D., Yang Q. (2023). Structural characteristics and gas sensing response of V_2_O_5_ nanorod thinfilms deposited by hot filament CVD. Sens. Actuators B Chem..

[16] Shrivathsa V., Shetty S.S., Bhat S., Jayarama A., Pinto R. (2022). Effect of precursor dilution solvents on the growth of V_2_O_5_ thin films using spray pyrolysis. Mater. Today Proc..

[17] Wang Y., Liang F., Zou Z., Zhang S., Jia S., Nong J., Zheng R., Song L. (2024). V_2_O_5_ layered nanofiber as an advanced cathode material in lithium ion batteries. J. Mater. Sci. Mater. Electron..

[18] Liu H., Liang X., Jiang T., Zhang Y., Liu S., Wang X., Fan X., Huai X., Fu Y., Geng Z. (2022). High-performance self-doped V4+-V_2_O_5_ ion storage films grown in situ using a novel hydrothermal-assisted sol-gel composite method. Electrochim. Acta.

[19] Bhosale M., Thangarasu S., Magdum S.S., Jeong C., Oh T.-H. (2024). Enhancing the electrocatalytic performance of vanadium oxide by interface interaction with rGO and NiO nanostructures for electrochemical water oxidation. Int. J. Hydrogen Energy.

[20] Kashif M., Thangarasu S., Murugan N., Magdum S.S., Kim Y.A., Kurkuri M., Oh T.-H. (2024). Interatomic interaction of 2D crumpled V_2_O_5_ nanosheets layered with Ni-MOF as a bifunctional electrocatalyst for overall water splitting and supercapacitor applications. J. Energy Storage.

[21] Ganesan R., Xavier J.R. (2024). Fabrication of polythiophene/graphitic carbon nitride/V_2_O_5_ nanocomposite for high-performance supercapacitor electrode. Mater. Sci. Eng. B.

[22] Haldar K.K., Ahmed I., Biswas R., Mete S., Patil R.A., Ma Y.-R. (2023). Efficient MoS_2_/V_2_O_5_ electrocatalyst for enhanced oxygen and hydrogen evolution reactions. Electrocatalysis.

[23] Munawar T., Bashir A., Iqbal F., Rafaqat M., Guo C., Zhiani M., Zhao C., Tong Y., Khan S.A., Koc M. (2025). g-C_3_N_4_-modulated CdS-V_2_O_5_/g-C_3_N_4_ nanosheets as efficient alkaline OER and HER electrocatalyst for water-splitting. Fuel.

[24] Varghese A., Sunajadevi K.R.P., Pinheiro D., Saravanakumar B., Selvaraj M. (2025). Advancing energy production and storage: Polypyrrole/V_2_O_5_/MnO_2_ composite as a high-performance electrocatalyst. Int. J. Hydrogen Energy.

[25] Alhawa D.A., Badreldin A., El-Ghenymy A., Hassan N., Wubulikasimu Y., Elsaid K., Abdel-Wahab A. (2024). Theoretical and experimental investigations of vanadium pentoxide–based electrocatalysts for the hydrogen evolution reaction in alkaline media. Emergent Mater..

[26] Yang Z., Xie X., Zhang Z., Yang J., Yu C., Dong S., Xiang M., Qin H. (2022). NiS_2_@ V_2_O_5_/VS_2_ ternary heterojunction for a high-performance electrocatalyst in overall water splitting. Int. J. Hydrogen Energy.

[27] Irshad A., Zulfiqar S., Alothman Z.A., Shakir I., Warsi M.F., Cochran E.W. (2024). Synergy of zero-dimensional carbon dots decoration on the one-dimensional architecture of Ag-doped V_2_O_5_ for supercapacitor and overall water-splitting applications. Fuel.

[28] Vinodh R., Deviprasath C., Gopi C.V.M., Kummara V.G.R., Atchudan R., Ahamad T., Kim H.-J., Yi M. (2020). Novel 13X Zeolite/PANI electrocatalyst for hydrogen and oxygen evolution reaction. Int. J. Hydrogen Energy.

[29] Tadesse M.G., Ahmmed A.S., Lübben J.F. (2024). Review on conductive polymer composites for supercapacitor applications. J. Compos. Sci..

[30] Sumdani M.G., Islam M.R., Yahaya A.N.A., Safie S.I. (2022). Recent advancements in synthesis, properties, and applications of conductive polymers for electrochemical energy storage devices: A review. Polym. Eng. Sci..

[31] Vinodh R., Babu R.S., Sambasivam S., Gopi C.V.M., Alzahmi S., Kim H.-J., de Barros A.L.F., Obaidat I.M. (2022). Recent advancements of polyaniline/metal organic framework (PANI/MOF) composite electrodes for supercapacitor applications: A critical review. Nanomaterials.

[32] Shah S.S., Oladepo S., Ehsan M.A., Iali W., Alenaizan A., Siddiqui M.N., Oyama M., Al-Betar A.R., Aziz M.A. (2024). Recent progress in polyaniline and its composites for supercapacitors. Chem. Rec..

[33] Meng X., Hou L., Jin H., Li W., Wang S., Wang Z., An J., Wen C., Ji G., Xu X. (2023). Study on corrosion protection properties of PANI/ZnO/Zn/Graphene coating on aluminum alloy. Diam. Relat. Mater..

[34] Eswaran M., Rahimi S., Pandit S., Chokkiah B., Mijakovic I. (2023). A flexible multifunctional electrode based on conducting PANI/Pd composite for non-enzymatic glucose sensor and direct alcohol fuel cell applications. Fuel.

[35] Chen M., Li M., Gao Y., He S., Zhan J., Zhang K., Huo Y., Zhu J., Zhou H., Fan J. (2024). Flexible and robust core–shell PANI/PVDF@ PANI nanofiber membrane for high-performance electromagnetic interference shielding. Nano Lett..

[36] Razzaq K., Gassoumi A., Abo-Dief H.M., Lmami M., Kumar A. (2025). Improvement in catalytic performance of CuFe_2_O_4_/PANI nanocomposite for robust water splitting. Electrochim. Acta.

[37] Fareed S., Alharbi F., Drissi N., Abo-Dief H.M., Gassoumi A., Kumar A. (2026). Hydrothermally fabricated perovskite-type composite (NiMnO_3_/PANI), an effective electrocatalyst for hydrogen evolution reaction (HER). Mater. Sci. Semicond. Process..

[38] Faiz A., Safra I., Aldhafeeri T.R., Ali S.K., Kumar A. (2025). Spinel-type nickel iron oxide anchored on conducting polymer PANI: A novel electrocatalyst for hydrogen evolution reaction (HER). Chem. Phys..

[39] Zhang J., Li Y., Wang Z., Wang Y., Wang F., Chen M. (2020). Three-dimensionally hierarchical NiCoP@ PANI architecture for high-performance hydrogen evolution reaction. Nanotechnology.

[40] Zahra R., Alrowaily A.W., Alotaibi B., Gassoumi A., Alyousef H.A., Mirza H., Kumar A., Ali M. (2025). Improving performance of CoMnO_3_ with PANI for alkaline hydrogen evolution reaction. Inorg. Chem. Commun..

[41] Rasool N., Alqarni A.S., Ahmad K., Al-Sehemi A.G., Henaish A., Aman S. (2024). Enhancement of BaCeO_3_ OER performance by generating a nanohybrid with gCN for electrochemical water splitting. Int. J. Hydrogen Energy.

[42] Guo J., Wei Z., Wang K., Zhang H. (2021). Synergistic coupling of CoFe-layered double hydroxide nanosheet arrays with reduced graphene oxide modified Ni foam for highly efficient oxygen evolution reaction and hydrogen evolution reaction. Int. J. Hydrogen Energy.

[43] Nandana A.B., Anand A.C., George S.M., Rakhi R.B. (2025). Electrochemical Investigation of V_2_O_5_ as a High-Performance Material for Supercapacitors and Electrocatalysis. ACS Appl. Electron. Mater..

[44] Zheng X., Qin M., Ma S., Chen Y., Ning H., Yang R., Mao S., Wang Y. (2022). Strong oxide-support interaction over IrO_2_/V_2_O_5_ for efficient pH-universal water splitting. Adv. Sci..

[45] Narayanasamy S., Jayaprakash J. (2021). Carbon cloth/nickel cobaltite (NiCo_2_O_4_)/polyaniline (PANI) composite electrodes: Preparation, characterization, and application in microbial fuel cells. Fuel.

[46] Dang Q., Sun Y., Wang X., Zhu W., Chen Y., Liao F., Huang H., Shao M. (2019). Carbon dots-Pt modified polyaniline nanosheet grown on carbon cloth as stable and high-efficient electrocatalyst for hydrogen evolution in pH-universal electrolyte. Appl. Catal. B Environ..

[47] Magdum S.S., Bhosale M., Palanisamy G., Selvakumar K., Thangarasu S., Oh T.H. (2025). Engineering heterojunction of multi-morphologies and bifunctional hybrid rGO-V_2_O_5_ embedded CeO_2_ nanostructures for robust visible-light-driven dye degradation and supercapacitor. J. Taiwan Inst. Chem. Eng..

[48] Ashraf I., Ahmad S., Nazir F., Dastan D., Shi Z., Garmestani H., Iqbal M. (2022). Hydrothermal synthesis and water splitting application of d-Ti3C2 MXene/V2O5 hybrid nanostructures as an efficient bifunctional catalyst. Int. J. Hydrogen Energy.

[49] Tamboli M.S., Patil S.A., Tamboli A.M., Patil S.S., Truong N.T.N., Lee K., Praveen C., Shrestha N.K., Park C., Kale B.B. (2022). Polyaniline-wrapped MnMoO_4_ as an active catalyst for hydrogen production by electrochemical water splitting. Dalton Trans..

[50] Chinnadurai D., Rajendiran R., Li O.L., Prabakar K. (2021). Mn-Co bimetallic phosphate on electrodeposited PANI nanowires with composition modulated structural morphology for efficient electrocatalytic water splitting. Appl. Catal. B Environ..

[51] Mathew S., Sim J.-H., Rajmohan R., Li O.L., Cho Y.-R. (2022). Defect-rich CoMoS nanosheets on PANI nanowires as excellent hybrid electrocatalyst for water splitting. Electrochim. Acta.

[52] Myasoedova T.N., Nedoedkova O.V., Kalusulingam R., Popov Y.V., Mikheykin A.S., Konstantinov A.S., Li Z., Mikhailova T.S., Shmatko V.A., Yalovega G.E. (2024). Fabrication of Ni-Polyaniline/Graphene Oxide Composite Electrode with High Capacitance and Water Splitting Activity. ChemPhysChem.

[53] Wen L., Li K., Liu J., Huang Y., Bu F., Zhao B., Xu Y. (2017). Graphene/polyaniline@ carbon cloth composite as a high-performance flexible supercapacitor electrode prepared by a one-step electrochemical co-deposition method. RSC Adv..

[54] Wang X., Wang Z., Jia B., Li C., Sun S., Hu S. (2024). Concerted proton-coupled electron transfer promotes NiCoP nanowire arrays for efficient overall water splitting at industrial-level current density. Chem. Eng. J..

[55] Meng L., Shang S., Liu S., Zhang L., Tang Q., Wang H., Wang F., Li C., Sun Y., Wu H. (2024). Cooperative effects between NiMo alloy enable highly efficient for all-pH-value and alkaline seawater hydrogen evolution. Appl. Catal. B Environ. Energy.

[56] Qin M., Ma G., Tan W., Fan Z., Xin X. (2023). Constructing an n–n junction between CoFe-LDH and NiCoP as bifunctional electrocatalysts for efficient overall water splitting. Inorg. Chem. Front..

[57] Zeng L., Sun K., Wang X., Liu Y., Pan Y., Liu Z., Cao D., Song Y., Liu S., Liu C. (2018). Three-dimensional-networked Ni_2_P/Ni_3_S_2_ heteronanoflake arrays for highly enhanced electrochemical overall-water-splitting activity. Nano Energy.

[58] Qu D. (2004). The study of the proton diffusion process in the porous MnO_2_ electrode. Electrochim. Acta.

[59] Xu T., Lu Z., Yu L., Xin W., Sun J., Zhou H., Jiao Y., Wang X., Lv C. (2025). Heterointerface engineering of vanadium oxide/cobalt nitride as efficient electrocatalysts for alkaline overall water splitting. Chem. Commun..

[60] El Maati L.A., Alomar M., Al-muhydeb L.B., Arifein W.U., Hassan R.U., Batool A. (2026). Development of an electrode material using a composite of perovskite oxide (NdCoO_3_) with PANI for effective HER. Solid State Commun..

